# Dopamine Regulates Angiogenesis in Normal Dermal Wound Tissues

**DOI:** 10.1371/journal.pone.0025215

**Published:** 2011-09-20

**Authors:** Saurav Shome, Tapasi Rana, Subhalakshmi Ganguly, Biswarup Basu, Sandipan Chaki Choudhury, Chandrani Sarkar, Debanjan Chakroborty, Partha Sarathi Dasgupta, Sujit Basu

**Affiliations:** 1 Department of Signal Transduction and Biogenic Amines, Chittaranjan National Cancer Institute, Kolkata, India; 2 Department of Pathology, Ohio State University, Columbus, Ohio, United States of America; 3 Dorthy M. Davis Heart and Lung Research Institute, Ohio State University, Columbus, Ohio, United States of America; 4 Arthur G. James Comprehensive Cancer Center, Ohio State University, Columbus, Ohio, United States of America; Children's Hospital Boston & Harvard Medical School, United States of America

## Abstract

Cutaneous wound healing is a normal physiological process and comprises different phases. Among these phases, angiogenesis or new blood vessel formation in wound tissue plays an important role. Skin is richly supplied by sympathetic nerves and evidences indicate the significant role of the sympathetic nervous system in cutaneous wound healing. Dopamine (DA) is an important catecholamine neurotransmitter released by the sympathetic nerve endings and recent studies have demonstrated the potent anti-angiogenic action of DA, which is mediated through its D_2_ DA receptors. We therefore postulate that this endogenous catecholamine neurotransmitter may have a role in the neovascularization of dermal wound tissues and subsequently in the process of wound healing. In the present study, the therapeutic efficacy of D_2_ DA receptor antagonist has been investigated for faster wound healing in a murine model of full thickness dermal wound. Our results indicate that treatment with specific D_2_ DA receptor antagonist significantly expedites the process of full thickness normal dermal wound healing in mice by inducing angiogenesis in wound tissues. The underlined mechanisms have been attributed to the up-regulation of homeobox transcription factor HoxD3 and its target α5β1 integrin, which play a pivotal role in wound angiogenesis. Since D_2_ DA receptor antagonists are already in clinical use for other disorders, these results have significant translational value from the bench to the bedside for efficient wound management along with other conventional treatment modalities.

## Introduction

Angiogenesis is an important process of new blood vessel formation that occurs in the body, both in health and in diseases. This process is further controlled by precise balance between pro- and anti-angiogenic factors in different normal physiological conditions like wound healing. Shifts in the finely tuned equilibrium between angiogenic stimulators and inhibitors that regulate angiogenesis lead to either excessive or insufficient angiogenesis, thereby causing many angiogenesis-dependent diseases, including cancer and other diseases such as atherosclerosis, age-related macular degeneration and rheumatoid arthritis [1].

Reports from our laboratory have shown that peripheral endogenous neurotransmitter dopamine by acting through its D_2_ DA receptors present in the endothelial cells (ECs) can significantly suppress vascular permeability factor/vascular endothelial growth factor (VEGF/VPF) induced tumor angiogenesis by inhibiting phosphorylation of vascular endothelial growth factor receptor 2 (VEGFR2), the principal VEGF receptor mediating the angiogenic effects of VEGF, focal adhesion kinase (FAK) and mitogen-activated protein kinase (MAPK) [2]–[5]. Moreover, DA can also suppress neovascularization in tumors by inhibiting mobilization of endothelial progenitor cells (EPCs) from the bone marrow to tumor vascular bed via DA D_2_ receptor-mediated inhibition of matrix metalloproteinase 9 (MMP-9) synthesis and ERK-1/ERK-2 signaling pathways in these cells [6]. These studies have conclusively demonstrated DA as a novel endogenous inhibitor of angiogenesis in malignant tumors.

In contrast to tumor angiogenesis, neovascularization in wound tissue is a normal physiological process essential for the regeneration of damaged tissues by formation of new blood vessels to maintain tissue viability, provide nutrients, and oxygen supply to the growing tissues, thereby aiding in the formation of provisional wound matrix or granulation tissue [7]–[11]. However, the regulatory role of DA, if any, in this process of physiological angiogenesis during wound tissue repair is not yet known. Dermal tissues are richly innervated by sympathetic nerves and recent reports indicate important role of these nerves in cutaneous wound healing [12]–[18]. Furthermore, dopamine is an important neurotransmitter in the sympathetic nervous system and DA has been established as an endogenous inhibitor of angiogenesis [2]–[6], [12]–[14]. Because DA mediates its anti-angiogenic effects by acting through its D_2_ receptors present in endothelial cells [2], we therefore investigated whether treatment with D_2_ DA receptor antagonist can stimulate angiogenesis in wound tissues to expedite the process of healing in a murine model of full thickness dermal wound.

## Results

### Treatment with specific D_2_ DA receptor antagonist accelerates dermal wound healing in mice

**Figure 1:**
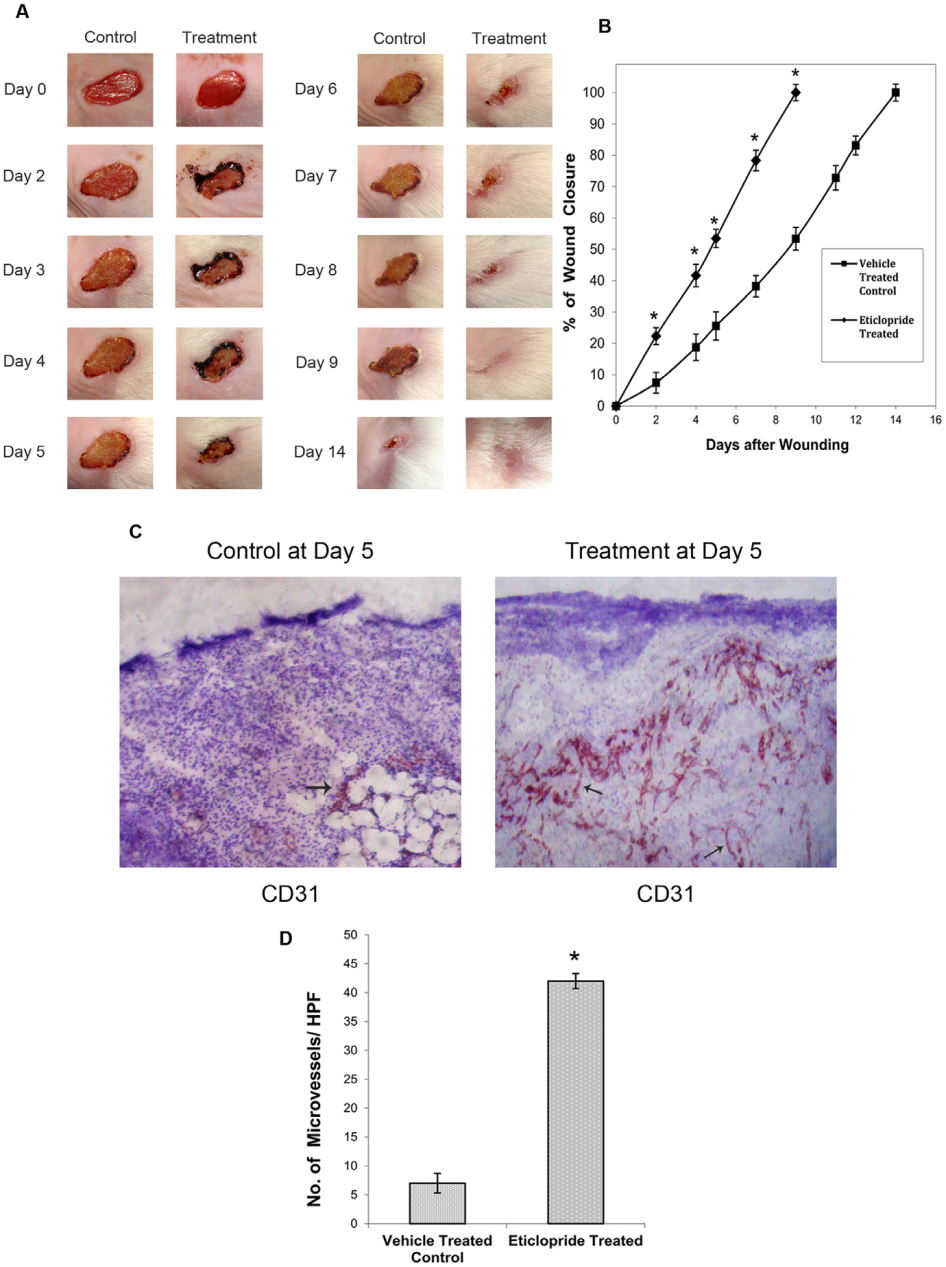
Effect of eticlopride treatment on macroscopic aspect of wound closure and formation of new blood vessels in wound bed. (**A**) The treatment with eticlopride, a specific D_2_ dopamine receptor antagonist; 10 mg/kg/4 days/i.p. significantly accelerated the rate of wound healing with resurfacing occurring by 9 days whereas complete wound closure in controls occurred on day 14. The control group received similar volume of normal saline only. (**B**) Wound closure analysis. In treatment group of mice eticlopride significantly accelerated wound closure compared to vehicle treated control (each group, n = 6; *, P<0.05). Time to wound closure was defined as the time until the re-epithelialization process was complete and the wound bed was filled with new tissues. The percentage of wound closure was calculated as: (area of original wound − area of actual wound) x100/area of original wound and were measured by analyzing images using an image analysis program (ImageJ, NIH). (**C**) Immunohistochemical staining of CD31, a specific endothelial cell surface marker to enumerate the number of microvessels. The figure shows significantly greater number of microvessels (reddish brown in color) in wound tissue sections of eticlopride treated mice in comparison to vehicle treated controls. Original magnifications, × 100. (**D**) Graphical representation shows significantly higher number of microvessels in eticlopride treated groups when compared to vehicle treated controls at day 5, 24 hours after completion of treatment schedule. Microvessel density was measured by counting the number of microvessels in 10 randomly chosen high power microscopic fields within the sections,*, P<0.05). Results are representative of six separate experiments each yielding similar results.

As angiogenesis is critical to successful wound repair and DA is an endogenous inhibitor of angiogenesis [2]–[6], the therapeutic efficacy of specific D_2_ DA receptor antagonist eticlopride *in vivo* was evaluated for faster wound healing in a murine model of full thickness dermal wound. Our results indicated that treatment with eticlopride (10 mg/kg/4 day i.p.), significantly improved the rate of wound healing in normal Swiss mice with resurfacing of intact, new skin occurring by 9 days (Fig. 1A), whereas the full thickness dermal wounds on the back of vehicle treated control Swiss mice required 14 days for complete closure. These results thus indicated that treatment with eticlopride significantly sped up (p<0.05) the healing process in wound bearing normal Swiss mice than vehicle treated control mice. The total surface area of the wound also decreased significantly after completion of the treatment on day 4 in comparison to saline treated controls (Fig. 1B). On day 5, the percent of wound closure was 53.4% in treated groups versus 25.5% in control groups, whereas it was 78.3% in treated versus 38.2% in controls on day 7 (Fig. 1B). Also the mean wound size of the treated group was always significantly smaller at all time points (p<0.05) than untreated groups from day 2 until the day of complete healing (Fig. 1B). Like eticlopride, treatment with domperidone, another D_2_ DA receptor specific antagonist also significantly accelerated the rate of wound healing in murine model of full thickness dermal wounds (data not shown). However, no significant changes were observed in the rate of wound healing when other dopamine receptor antagonists (D_1_, D_3_, D_4_ and D_5_) were used (data not shown). This data confirmed that the action of DA was specific and was through its D_2_ receptors.

### Significantly increased angiogenesis in wound bed of specific D_2_ DA receptor antagonist treated group than vehicle treated controls

As angiogenesis plays a pivotal role in wound healing and endogenous DA by acting through its D_2_ receptors acts as a potent inhibitor of angiogenesis, we therefore investigated whether the eticlopride mediated faster wound healing was associated with significantly increased angiogenesis in wound tissues. At day 5 after the completion of 4 day treatment schedule of eticlopride, the number of microvessels was significantly higher (p<0.05) in wound beds of eticlopride treated mice than vehicle treated controls (Fig. 1C and 1D). This significantly enhanced angiogenesis in wound tissues also correlated well with the faster wound healing in eticlopride treated mice. Microvessel density was calculated by counting the number of CD31 positive cells after performing immunohistochemistry of frozen wound tissue sections [6].

### Increased expression of HoxD3, a regulator of angiogenesis and its target gene α5β1 in wound bed following treatment with specific D_2_ DA receptor antagonist

Among the different genes regulating the process of angiogenesis, HoxD3, a homeobox transcription factor, plays a critical role in regulating neovascularization in wounds [19]–[21]. HoxD3 is a member of the homeobox (Hox) family of master transcription factors that are expressed during normal embryogenesis, skin development and during fetal wound healing [22]–[24]. Furthermore, HoxD3 can modulate the expression of number of genes, among which α5β1 integrin is well associated with angiogenesis [20], [25], [26]. Therefore, the mechanism of D_2_ dopamine receptor antagonist eticlopride mediated increased angiogenesis in wound tissue as observed in the present study was explored by determining the expression of HoxD3 and α5β1 integrin in wound bed.

**Figure 2:**
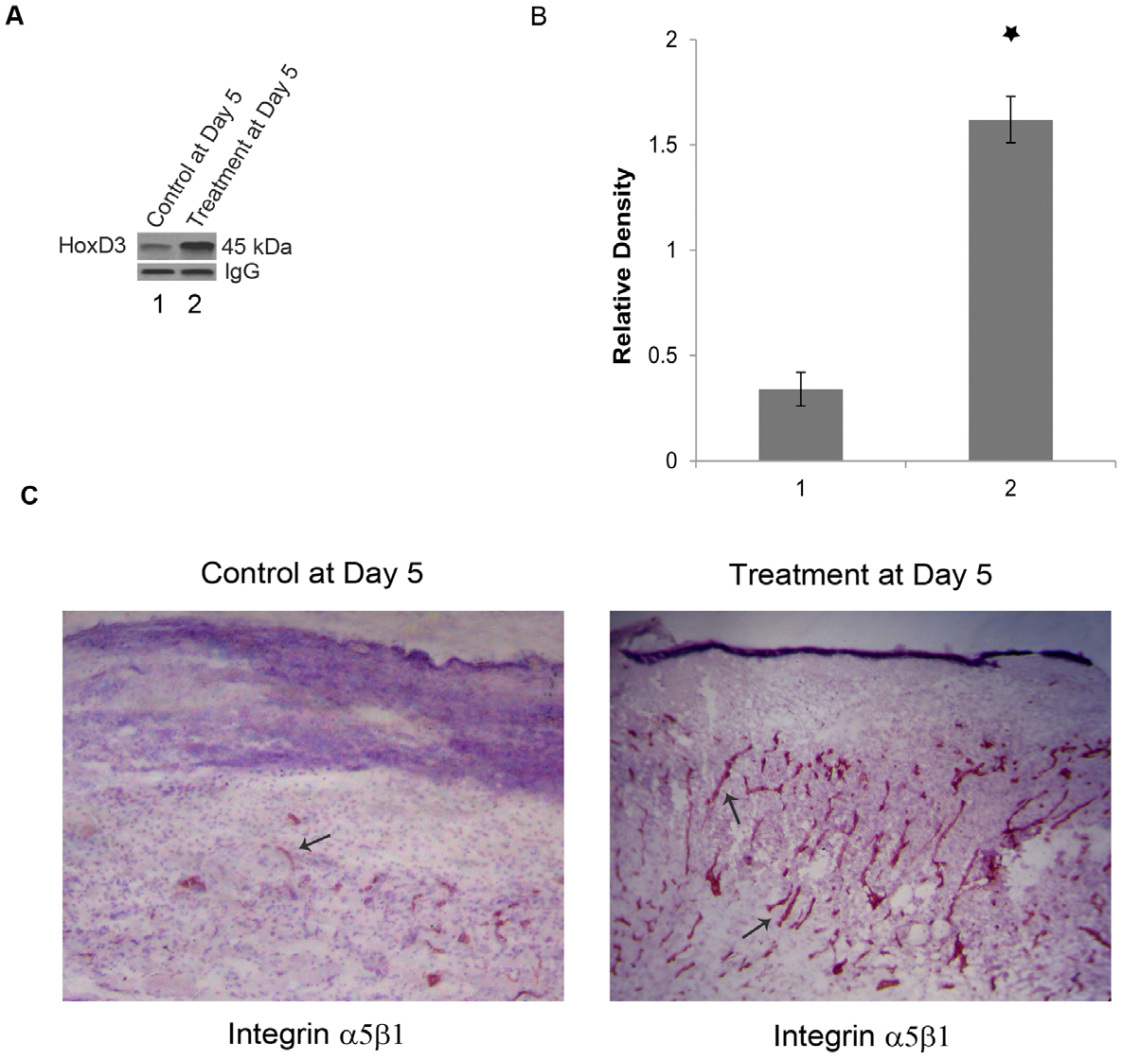
Effect of eticlopride treatment on expression of HoxD3 and its target α5β1 integrin in cutaneous wound tissue of mice. (**A**) Immunoprecipitation followed by immunoblot analysis of HoxD3 expression in wound tissues of both eticlopride and saline treated wound bearing mice at day 5 post wounding. The immunoblot analysis shows significantly higher expression of HoxD3 protein in wound tissues of eticlopride treated mice than vehicle treated controls at 5^th^ day after creation of wounds. (**B**) The bar graphs represent the density of each HoxD3 protein band relative to the IgG expression as quantified by ImageJ (NIH), *, P<0.05. (**C**) Immunohistochemical analysis of the expression of α5β1 integrin in cutaneous wound tissues of both control and eticlopride treated mice at day 5 post wounding. Frozen sections were immunostained with anti-α5β1 integrin antibodies followed by biotin conjugated secondary antibodies. The sections were stained using ABC staining kit and Nova-Red substrate solution to develop color. Significantly more areas of wound bed show positive staining (reddish brown color) for α5β1 integrin following eticlopride treatment. Original magnifications, x 100. Results are representative of six separate experiments each yielding similar results.

Our results indicate that eticlopride treatment, which accelerated wound tissue neovascularization had significant positive regulatory effect on the expression of this master transcription factor (HoxD3) in wound tissue as evident from immunoblot analysis at day 5 post wounding (24 hours after completion of 4 day schedule of eticlopride treatment) (Fig. 2A and 2B). In addition, on day 5, following completion of eticlopride treatment, the expression of α5β1 integrin, target gene of the transcription factor HoxD3 was also significantly up-regulated in these wound tissues as determined by immunohistochemistry (Fig. 2C).

### DA inhibits the expression of HoxD3 and its target genes α5 and β1 integrins in HUVEC

**Figure 3:**
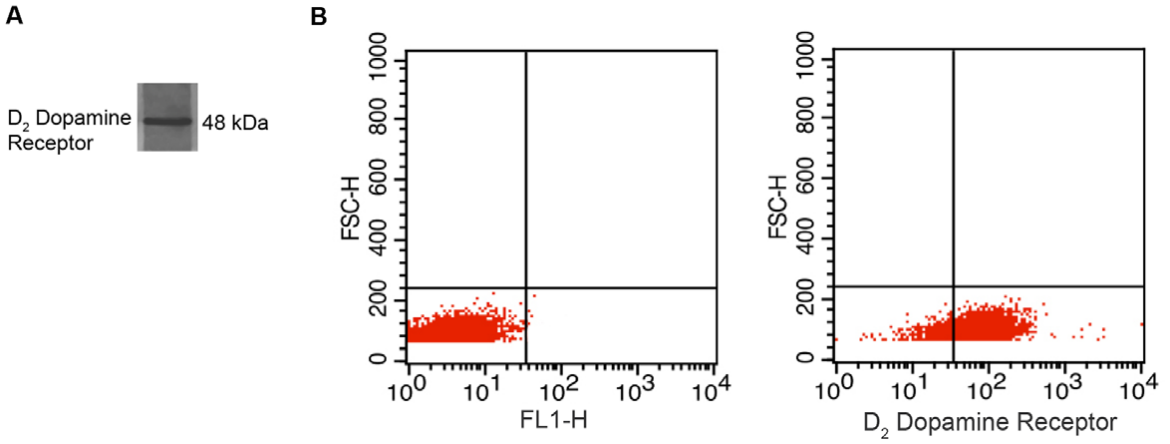
D_2_ Dopamine receptors are present on the surface of human umbilical vein endothelial cells. (**A**) Immunoblot shows presence of D_2_ dopamine receptors in HUVEC. (**B**) Flow cytometric analysis of D_2_ dopamine Receptors in HUVEC. Over 81% cells of the total HUVEC population express D_2_ dopamine receptors on their surfaces as evident from the lower right quadrant.

Endothelial cells are principal cellular components of the blood vessels and proliferation and migration of these cells play a central role in angiogenesis [1], [7]. Our *in vivo* results indicated increased expressions of HoxD3 transcription factor along with α5β1 integrin in wound tissues following eticlopride treatment, which in turn was associated with increased angiogenesis in wound bed. It was also reported that in endothelial cells, up-regulation of HoxD3 and its target gene α5β1 integrin were closely associated with angiogenic processes like proliferation and migration of these cells following exposure to growth factors [20], [25]. Among the different growth factors that regulate wound angiogenesis, VEGF plays a critical role in inducing angiogenesis in wound tissues. Furthermore VEGF is thought to regulate different processes like vascular permeability, migration and proliferation of endothelial cells during wound repair [27]–[29]. Therefore, experiments were designed *in vitro* to examine the direct effects of DA on the expressions of HoxD3 and α5β1 integrin in endothelial cells following treatment with VEGF. Since dermal tissues are richly supplied with sympathetic nerves and because 1 µM of DA is present in the extracellular fluid surrounding neural synapses [30], we therefore used this concentration of DA for our *in vitro* experiments. HUVEC were especially selected because these cells express both HoxD3 and D_2_ DA receptors [2], [20], [31]. It is also to be noted here that murine dermal angiogenic endothelial cells express D_2_ DA receptors [2]. Immunoblot of HUVEC showed presence of D_2_ DA receptors in these cells (Fig. 3A). This was further confirmed by flow cytometry analysis which revealed that over 81% cells of the total HUVEC population express D_2_ dopamine receptors on their surfaces (Fig. 3B).

**Figure 4:**
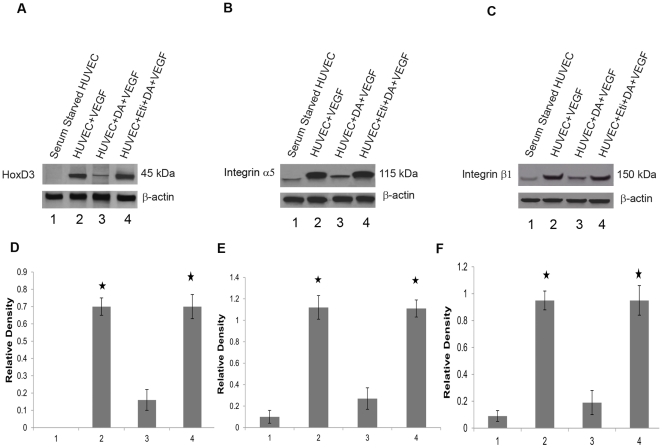
Dopamine through its D_2_ receptors can significantly downregulate VEGF induced expressions of HoxD3 and its target genes α5 and β1 integrins in HUVEC. (**A, B and C**) Western blot analysis of the effect of dopamine on VEGF induced expressions of HoxD3 and its target genes α5 and β1 integrins in HUVEC. Lane 1: Serum starved Human Umbilical Vein Endothelial Cells show no expression of HoxD3. However expression of both α5 and β1 integrins was observed. Lane 2: Cells stimulated with VEGF (10 ng/ml) show significant expression of HoxD3 and both the integrins after 8 hours of stimulation. Lane 3: Cells pretreated with 1 µM dopamine (concentration of DA found in synaptic clefts) 5 minutes before being exposed to VEGF (10 ng/ml) show significantly down-regulated VEGF induced expression of HoxD3 and both α5 and β1 integrins compared with VEGF treated controls. Lane 4: Cells treated with 100 µM eticlopride followed by dopamine and VEGF. Pre-treatment with eticlopride abrogated dopamine-induced down-regulation of HoxD3 and its target α5 and β1 integrin expression in HUVEC. β-actin was used as loading controls. Results are representative of six separate experiments each yielding similar results. (**D, E and F**) The bar graphs represent the density of each protein band relative to the β-actin expression. These have been quantified by ImageJ (NIH), *, P<0.05.

Although serum starved HUVEC did not show any expression of HoxD3, significant expressions of both HoxD3 and its target genes α5 and β1 integrins were observed 8 hrs after treatment with VEGF at a concentration of 10ng/ml. In contrast, when exposed to DA (1 µM), the expressions of both HoxD3 and its target genes α5 and β1 integrins were significantly down-regulated. However, pre-treatment with specific D_2_ DA receptor antagonist eticlopride abrogated the effects of DA (Fig. 4A-4C) thus further indicating that inhibition of this growth factor induced HoxD3 expression by DA was mediated through its D_2_ receptors. These results corroborate with our *in vivo* experiments where eticlopride treatment was associated with increased expressions of HoxD3 and α5β1 integrin along with stimulation of angiogenesis in wound tissue. These results also for the first time demonstrated that endogenous DA acts as a negative regulator of wound angiogenesis and treatment with D_2_ DA receptor specific antagonist abrogates this negative effect of DA as increased angiogenesis was observed in the wound tissues of eticlopride treated animals.

## Discussion

Skin is richly innervated by sympathetic nerves and there are now several reports which indicate that these nerves can regulate healing of cutaneous wounds [12], [15]–[18]. It is also now well established that angiogenesis plays a central role in cutaneous wound tissue repair by influencing different facets of wound healing [9]–[11]. DA is one of the catecholamine neurotransmitters released from the sympathetic nerve endings [12]–[14] and we had earlier demonstrated DA to be an endogenous inhibitor of tumor angiogenesis [2]–[6].

Our present results have shown that treatment with eticlopride, a specific D_2_ DA receptor antagonist significantly accelerates angiogenesis in wound tissues, thereby inducing faster healing of full thickness dermal wounds in normal mice. As angiogenesis induces rapid and successful wound healing [19], [27], [32], therefore, it can be suggested that eticlopride by augmenting neovascularization induces an ambient microenvironment in the wound bed favorable for implementing the other major steps required for wound healing.

Moreover recent studies have demonstrated the important roles of HoxD3, a member of homeobox (Hox) family of transcription factors, in wound angiogenesis [19]–[21]. The expression of HoxD3 is up-regulated in normal wound tissues, whereas its expression is decreased in poorly healing wounds of genetically diabetic (db/db) mice [19]–[21]. Interestingly, restoration of HoxD3 by gene transfer has been reported to accelerate diabetic wound healing by modulating the expressions of number of genes associated with wound angiogenesis [19]–[21], [25]–[26]. Therefore, to understand the molecular basis of this increased angiogenesis in wound bed following D_2_ DA receptor antagonist treatment, the expressions of HoxD3 and its principal target gene α5β1 integrin in wound tissues were examined. *In vivo* results revealed a direct correlation between significant up-regulation of HoxD3 and its target gene α5β1 integrin in wound tissues and wound neovascularization following eticlopride treatment. This present data also corroborates well with the previous observations by Boudreau and Varner demonstrating the regulatory role of the transcription factor HoxD3 in the expression of α5β1, an important initiator of angiogenesis [25]. It has been reported that HoxD3 binds directly to the promoters of integrin α and β subunits and induces their expressions in endothelial cells during angiogenesis, whereas knock down of HoxD3 by antisense treatment, significantly down-regulates the expression of α5β1 in endothelial cells [25]. As the results of our *in vivo* experiments had indicated increased expression of HoxD3 and its target α5β1 integrin in wound beds of mice following eticlopride treatment, further experiments were designed *in vitro* to confirm the *in vivo* results. Recent studies have indicated that growth factors can induce HoxD3 expression in endothelial cells, which in turn is associated with increased proliferation and migration of endothelial cells, a prerequisite for formation of new blood vessels [19], [20], [25]. Our *in vitro* experiments demonstrate that activation of D_2_ dopamine receptors significantly inhibited VEGF-induced expression of HoxD3 and its target genes α5 and β1 integrins in endothelial cells. In contrast, pre-treatment with eticlopride, abrogated this DA induced down-regulation of both HoxD3 and its target integrins. These *in vitro* results also correlated well and confirmed our *in vivo* results showing eticlopride induced increased expression of HoxD3 and its target integrins in wound tissues, thus indicating a novel association between endogenous DA and wound angiogenesis. However, angiogenesis in wound tissue is a complex process, involving multiple growth factors and cytokines, therefore besides influencing HoxD3 expression, the possibility of involvement of DA through its D_2_ receptors to interact with other growth factors and their down stream pro-angiogenic target genes cannot be ruled out.

Taken together, the information generated from the present study thus has shown for the first time that in addition to regulating pathological angiogenesis in tumors [5]–[6], DA through its D_2_ receptors can also negatively influence physiological angiogenesis in wounds and treatment with a specific D_2_ dopamine receptor antagonist can significantly increase wound angiogenesis and thereby, expedite the process of wound tissue repair. This novel information has immense translational value in respect to better and efficient wound management by using D_2_ DA receptor antagonists along with other conventional therapy for the repair of damaged wound tissues.

## Materials and Methods

### Experimental wound model

All animal experiments were performed after approval by the Institutional Animal Care and Use Committees. In the present investigation, the experiments were carried out in full thickness dermal wound bearing normal Swiss mice (4–6 weeks and weighing 22–25 g). The animals were anesthetized with intraperitoneal (i.p.) injection of 100 µl solution containing ketamine and xylazine mixture (2.215 and 0.175 mg, respectively; all from Sigma, St. Louis). The dorsal hair of the mouse was shaved and disinfected with an alcohol (70% ethanol) swab to prepare the back skin for generation of a standardized full-thickness dermal wound. Using an 8 mm dermal punch biopsy, two excisional wounds were created at the same cranial-caudal level on the dorso-medial back of each animal. At the end of the surgical procedure, cages were placed on a heating pad until mice fully recovered from anesthesia [33].

### Treatment schedule with eticlopride, a specific D_2_ DA receptor antagonist

After creation of wounds, the wound bearing mice were divided into two equal groups. Immediately after wounding, mice of the treatment group were given 10 mg/kg i.p. of eticlopride hydrochloride (Sigma, USA) in 300 µl normal saline and continued for four consecutive days with 24 hours interval [2]. The control group received similar volume of normal saline only.

### Wound Healing Studies

Wound closure was documented with a digital camera. Time to wound closure was defined as the time until the re-epithelialization process was complete and the wound bed was filled with new tissues. Wound area was measured by analyzing images using an image analysis program (ImageJ, NIH) by tracing the wound margin and calculating the pixel area. The pixel counts were then related to a circular filter paper having the same diameter as the original wound, which served as a reference for every wound healing image assessment. The measurements were performed in triplicate and mean values of consecutive tracings were computed and expressed as percentage of closure from the original wound. The percentage of wound closure was calculated as: (area of original wound − area of actual wound) x 100/area of original wound [33], [34].

### Examination of wound vascularity, HoxD3 and integrin expression

On day 5, after completion of eticlopride treatment for four consecutive days, the extent of wound tissue neovascularization and integrin expressions were examined in both vehicle treated controls and eticlopride treated mice. Wound tissues from both the groups were collected, washed in PBS and fixed in OCT compound at −20°C for 1 hour and 5 µm cryostat sections were cut from the mid-portion of the wounds with a cryomicrotome. Sections were taken onto Poly-L-Lysine coated glass slides and fixed in methanol at −20°C for 20 minutes and the slides were stored at −80°C until assayed. The sections were stained with anti-CD31 antibody (Goat anti-mouse IgG; R&D Systems, MN) or anti-integrin α5β1 antibody (Rat anti-mouse IgG; Millipore, MA) and biotin conjugated secondary antibodies (Rabbit anti-goat IgG; Millipore, MA and Goat anti-rat; BD Pharmingen, CA) and treated with ABC staining kit (Vector Laboratories, CA) and Nova-Red substrate solution (Vector Laboratories, CA) to develop color. Microvessel density (CD31 positive cells) in wound bed was measured by counting the number of microvessels in 10 randomly chosen high power microscopic fields within the sections [6].

For immunoblot analysis of HoxD3 in wound tissue, the entire wound, as well as a ∼2 mm margin of surrounding normal skin were excised, cut into small pieces with the help of a clean sharp razor blade, homogenized and the protein extracts from cells were immunoprecipitated with anti-HoxD3 antibody (Santa Cruz, CA) and immunoprecipitates were captured on protein A-agarose beads. The immunocomplexes were then subjected to SDS-PAGE and then transferred to polyvinyl difluoride membranes (Millipore, MA) and immunoblotted. Goat anti-rabbit IgG HRP conjugated (Santa Cruz, CA) was used as secondary antibody. Antibody reactive bands were detected by enzyme - linked chemiluminescence (Pierce IL) [4].

### Determination of D_2_ DA receptors in HUVEC by immunoblotting and flow cytometry

HUVEC (human umbilical vein endothelial cells) (HUV-EC-C; ATCC Number: CRL-1730) were cultured in ATCC formulated F-12K Medium (Catalog No. 30-2004). To make the complete growth medium, the following components were added to the base medium: 0.1mg/ml heparin; 0.03–0.05 mg/ml endothelial cell growth supplement (ECGS; Millipore, MA) & adjusted to a final concentration of 10% fetal bovine serum. The presence of D_2_ DA receptors in HUVEC were determined by both immunoblot and flow cytometry analysis.

*In vitro* expanded HUVEC were lysed and the protein extracts from cells were immunoprecipitated with mouse anti-D_2_ DA receptor antibody (Santa Cruz, CA) and immunoprecipitates were captured on protein A-agarose beads. Thereafter, the immunocomplexes were subjected to SDS-PAGE and then transferred to polyvinyl difluoride membranes (Millipore, MA) and immunoblotted. Goat anti-mouse IgG HRP conjugated (Santa Cruz, CA) was used as secondary antibody. Antibody reactive bands were detected by enzyme - linked chemiluminescence (Pierce, IL) [4].

Cultured HUVEC were further analyzed by flow cytometry (FACS Calibur; BD Biosciences) to determine the presence of D_2_ DA receptors in these cells. HUVEC were incubated with mouse anti-D_2_ DA receptor antibody, (Santa Cruz, CA). After incubation with the primary antibody, FITC conjugated rat anti-mouse IgG (eBioscience, CA) against the primary anti-D_2_ DA receptor antibody was also added. Initial analysis gates were designed to exclude dead cells and debris. Analyses were considered as informative when adequate numbers of events (after acquisition of 10000 cells per sample) were collected in the gated cells. Percentage of D_2_ DA receptor positive cells was finally determined after comparing them with matched isotype controls [6].

### Effects of DA on expressions of HoxD3 and its target genes α5 and β1 integrins in HUVEC

To determine effect of DA receptor activation on HoxD3 expression *in vitro*, HUVEC were serum-starved for 24 hours and then treated with 1 µM DA followed by addition of 10 ng/ml VEGF (Millipore, MA) 5 minutes later. After incubation for 8 hours, cells were collected for protein isolation [2].

The protein extracts from cells of various experimental groups were subjected to SDS-PAGE (non-reducing condition in case of α5 integrin only) separately for each of these proteins and blotted onto PVDF membranes (Millipore, MA). Primary antibody used in western blots were rabbit anti-HoxD3 IgG (Santa Cruz, CA), rabbit anti-Integrin α5 (Millipore, MA) and rabbit anti-Integrin β1 (Millipore, MA). Goat anti-rabbit IgG HRP conjugated (Santa Cruz, CA) was used as secondary antibody for the western blots [31].

### Statistical analysis

Data are means of at least 6 different experiments ± SEM. Student's t-test was used to analyze differences between groups. *p* value <0.05 was considered statistically significant [33], [34].
